# Animal agriculture exposures among Minnesota residents with zoonotic enteric infections, 2012–2016

**DOI:** 10.1017/S0950268819002309

**Published:** 2020-03-16

**Authors:** CA Klumb, JM Scheftel, KE Smith

**Affiliations:** Foodborne, Waterborne, Vectorborne, and Zoonotic Diseases Section, Infectious Disease Epidemiology, Prevention and Control Division, Minnesota Department of Health, St. Paul, MN, USA

**Keywords:** Agriculture, enterics, occupation-related infections, zoonoses

## Abstract

Prospective, population-based surveillance to systematically ascertain exposures to food production animals or their environments among Minnesota residents with sporadic, domestically acquired, laboratory-confirmed enteric zoonotic pathogen infections was conducted from 2012 through 2016. Twenty-three percent (*n* = 1708) of the 7560 enteric disease cases in the study reported an animal agriculture exposure in their incubation period, including 60% (344/571) of *Cryptosporidium parvum* cases, 28% (934/3391) of *Campylobacter* cases, 22% (85/383) of Shiga toxin-producing *Escherichia coli* (STEC) O157 cases, 16% (83/521) of non-O157 STEC cases, 10% (253/2575) of non-typhoidal *Salmonella enterica* cases and 8% (9/119) of *Yersinia enterocolitica* cases. Living and/or working on a farm accounted for 61% of cases with an agricultural exposure, followed by visiting a private farm (29% of cases) and visiting a public animal agriculture venue (10% of cases). Cattle were the most common animal type in agricultural exposures, reported by 72% of cases. The estimated cumulative incidence of zoonotic enteric infections for people who live and/or work on farms with food production animals in Minnesota during 2012–2016 was 147 per 10 000 population, *vs.* 18.5 per 10 000 for other Minnesotans. The burden of enteric zoonoses among people with animal agriculture exposures appears to be far greater than previously appreciated.

## Introduction

Food-production animals are a well-known source of zoonotic enteric pathogens, particularly *Campylobacter*, *Cryptosporidium parvum,* non-typhoidal *Salmonella enterica* (NTS) and Shiga toxin-producing *Escherichia coli* (STEC). This has been demonstrated in numerous outbreaks associated with agricultural settings and in case-control studies of sporadic infections with these pathogens [1–8]. Transmission can occur through direct contact with animals, or through indirect contact, which involves touching objects or environmental surfaces that have been contaminated by animal faeces [8–10].

There have been few studies on the proportion of zoonotic enteric pathogen infections that can be attributed to animal contact, as opposed to foodborne or other transmission routes. In agricultural workers and their families specifically, the burden of zoonoses acquired from contact with food production animals has been less well quantified than the burden of fatal and non-fatal injuries [11–15]. In the United States, the 2008 National Occupational Research Agenda (NORA) for Agriculture, Forestry and Fishing included surveillance to describe the nature and extent of occupational illnesses as a top priority in its first Strategic Goal [16]. This goal has remained largely unfulfilled in the last decade. Consequently, the 2018 NORA for Agriculture, Forestry and Fishing reiterated the need for improved surveillance, and called for research to better describe animal exposures and associated zoonotic infections [17].

Animal agriculture is important in Minnesota; the state is the top producer of turkeys in the United States and the number two producer of hogs, and ranks eighth in livestock production overall [18]. To help address the data gaps in zoonotic disease surveillance in agricultural populations, the Minnesota Department of Health (MDH) conducted long-term, prospective surveillance of common reportable enteric zoonotic pathogens to systematically ascertain and characterize case exposures to food animals. The study aims were to describe the frequency and burden of enteric zoonoses in agricultural workers, their families and others exposed to agricultural settings in Minnesota and to identify disease and setting-specific risk factors. This work was conducted as part of the Upper Midwest Agricultural Safety and Health Center (UMASH) [19], 1 of 11 National Institute for Occupational Safety and Health (NIOSH) Centers of Excellence in Agricultural Disease and Injury Research, Education and Prevention [20].

## Methods

The MDH has performed statewide, active, population-based surveillance for reportable enteric pathogens in Minnesota residents since 1996. In addition to reporting, clinical laboratories are required to submit isolates or clinical specimens from cases to the MDH Public Health Laboratory, where pathogens are confirmed and characterized. This study focused on cases with *Campylobacter*, *Cryptosporidium parvum*, NTS, STEC O157, non-O157 STEC (all serogroups) and *Yersinia enterocolitica* infections with specimen collection dates during 2012–2016.

MDH staff attempt to interview all Minnesota residents with a laboratory-confirmed reportable enteric infection. Standard pathogen-specific surveillance questionnaires include questions about symptoms, ill contacts, and food, water, travel and animal exposures during the exposure period (7 days for all pathogens except *Cryptosporidium* (14 days)). During the entire study period, the animal agriculture exposure questions in the surveillance interview were the same for all pathogens. Cases were asked if they lived on, worked on or visited a private farm, if they visited a petting zoo, educational exhibit, fair or other public venue with food animals during their exposure period, and if they had exposure to pet livestock or poultry (Supplementary Figure S1 available on the Cambridge Core website). Cases who answered yes were then asked if cattle, goats, sheep, pigs, chickens, turkeys or other animals were present. If food animals were present, cases were considered to have had exposure to an animal agriculture setting. Cases were also asked whether that exposure included direct contact with the animals they reported.

Cases who reported exposure to an animal agricultural setting were categorized into one of three tiers: Tier 1 – living and/or working on a farm; Tier 2 – visiting a private farm and, Tier 3 – visiting a fair, petting zoo, agritourism farm or other public animal agriculture venue. Cases who reported living on a hobby farm or a premise with backyard poultry were categorized as Tier 1. If multiple tiers were reported, a case was assigned to the highest risk tier, with Tier 1 considered the highest risk tier, followed by Tier 2, then Tier 3. This Tier risk ranking was established because historically in Minnesota there have been many more sporadic cases with the study pathogens who reported private farm exposures than who reported public animal contact venue exposures.

Cases who reported an animal agriculture exposure were re-interviewed using a tier-specific questionnaire to gather more detailed information about the animal type(s) involved, type of contact and specific exposures (Supplementary Figures S2, S3 and S4 available on the Cambridge Core website). Tier 1 settings were further categorized as either a ‘full-time’ or ‘part-time’ farm. A ‘full-time’ farm was defined as a farm that sold at least $1000 of product in the past year and a ‘part-time’ farm was one that sold <$1000 of product in the past year [21, 22]. Among Tier 1 cases, farm characteristics, job duties, time on farm prior to illness onset, amount of work/school missed due to illness, working knowledge of zoonotic diseases and practices in place to reduce infection risk were analysed.

This study focused on the burden of domestically acquired, sporadic (non-outbreak-associated) cases potentially due to animal agriculture exposures in Minnesota. Therefore, cases were excluded if they refused the original surveillance interview or could not be contacted, were part of an outbreak, travelled internationally during the exposure period, were infected with a *Cryptosporidium* strain not confirmed as *C. parvum*, or reported an animal agricultural exposure in a state other than Minnesota.

The proportion of cases reporting an animal agriculture exposure was examined by pathogen, tier and animal type. The demographics and clinical characteristics of cases with an animal agriculture exposure were described.

Cumulative disease incidence for the 5-year study period among people who live and/or work on farms with food production animals in Minnesota was estimated. The denominator used for this calculation (*n* = 70 871) was the number of hired and unpaid livestock and poultry workers and operators, plus the number of children <13 years of age living on a farm with livestock or poultry, in Minnesota. The number of workers and operators was determined using the 2012 Census of Agriculture, combined with North American Industry Classification System (NAICS) codes for beef cattle (112 111), cattle feedlots (112 112), dairy cattle and milk production (11 212), hog and pig (1122), poultry and egg (1123) and sheep and goats (1124) [21, 22]. The average population of children living on any type of farm during the study period was determined using American Community Survey (ACS) data from 2012–2016 [23]. The number of children living on livestock/poultry farms specifically was estimated based on the proportion of livestock/poultry farms *vs.* crop farms indicated in the 2012 Census of Agriculture.

The NAICS classifies establishments to the Animal Production and Aquaculture subsector only when animal production (i.e. value of animals for market) accounts for one-half or more of the establishment's total agricultural production. Because some crop farms also have food production animals, we also conducted a sensitivity analysis in which we included crop farm workers and operators, and children <13 years who live on a crop farm in the livestock worker/operator (plus children) denominator to provide a more conservative cumulative incidence estimate; this new denominator was *n* = 244 067. NAICS codes for oilseed and grain farming (1111); vegetable and melon farming (1112); fruit and tree nut farming (1113) and other crop farming (1119) were used to estimate the number of hired and unpaid crop farmers and operators in Minnesota [22]. ACS data from 2012–2016 were used to calculate the number of children [23].

Minnesota state population estimates for 2012–2016 were obtained through the Minnesota State Demographic Center [24]. Finally, the incidence of zoonotic enteric infections among those who lived and/or worked on a farm with food production animals (Tier 1) using both methods was compared to that of all other Minnesotans.

The number of outbreak cases of *Campylobacter*, *C. parvum*, NTS, STEC O157 and non-O157 STEC infection associated with an animal agriculture setting was compared to the number of sporadic cases with exposure to an animal agriculture setting. Additionally, the number of outbreak cases associated with an animal agriculture setting was compared to the number of outbreak cases associated with all other transmission routes (e.g. foodborne, waterborne, person-to-person). The source of data for these analyses was MDH outbreak records, and included cases from Minnesota-only outbreaks as well as Minnesota cases from multi-state outbreaks.

Data analyses were performed using SAS 9.4 (SAS Institute, Cary, NC, USA) statistical software. Univariate analyses were conducted; dichotomous variables were analysed using a *χ*^2^ test and continuous variables were analysed using ANOVA; a two-sided *P*-value <0.05 was considered statistically significant.

## Results

**Fig. 1.**
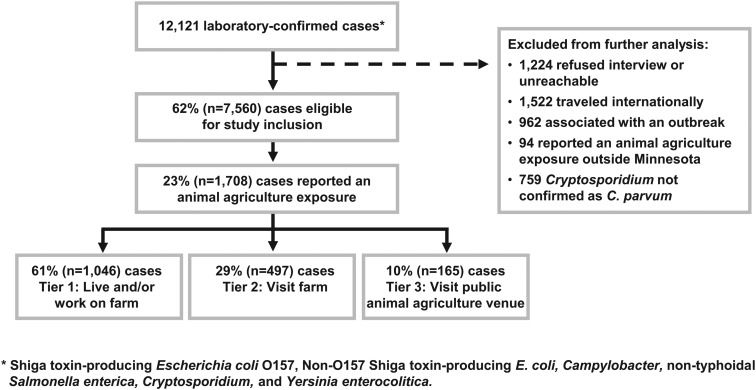
Flow diagram for enrolment of reportable zoonotic enteric disease cases into the study of animal agriculture exposures, Minnesota, 2012–2016.

From 2012 through 2016, 12121 laboratory-confirmed infections of *Campylobacter, Cryptosporidium,* NTS, STEC O157, non-O157 STEC and *Yersinia enterocolitica* were reported to MDH. After exclusions, 7560 (62%) cases were eligible for study inclusion (Fig. 1). Of these, 1708 (23%) reported an animal agriculture exposure in Minnesota during their exposure period; 1046 (61%) reported living and/or working on a farm (Tier 1), 497 (29%) reported visiting a private farm (Tier 2) and 165 (10%) reported visiting a public animal agriculture venue (Tier 3) (Fig. 1).

**Fig. 2.**
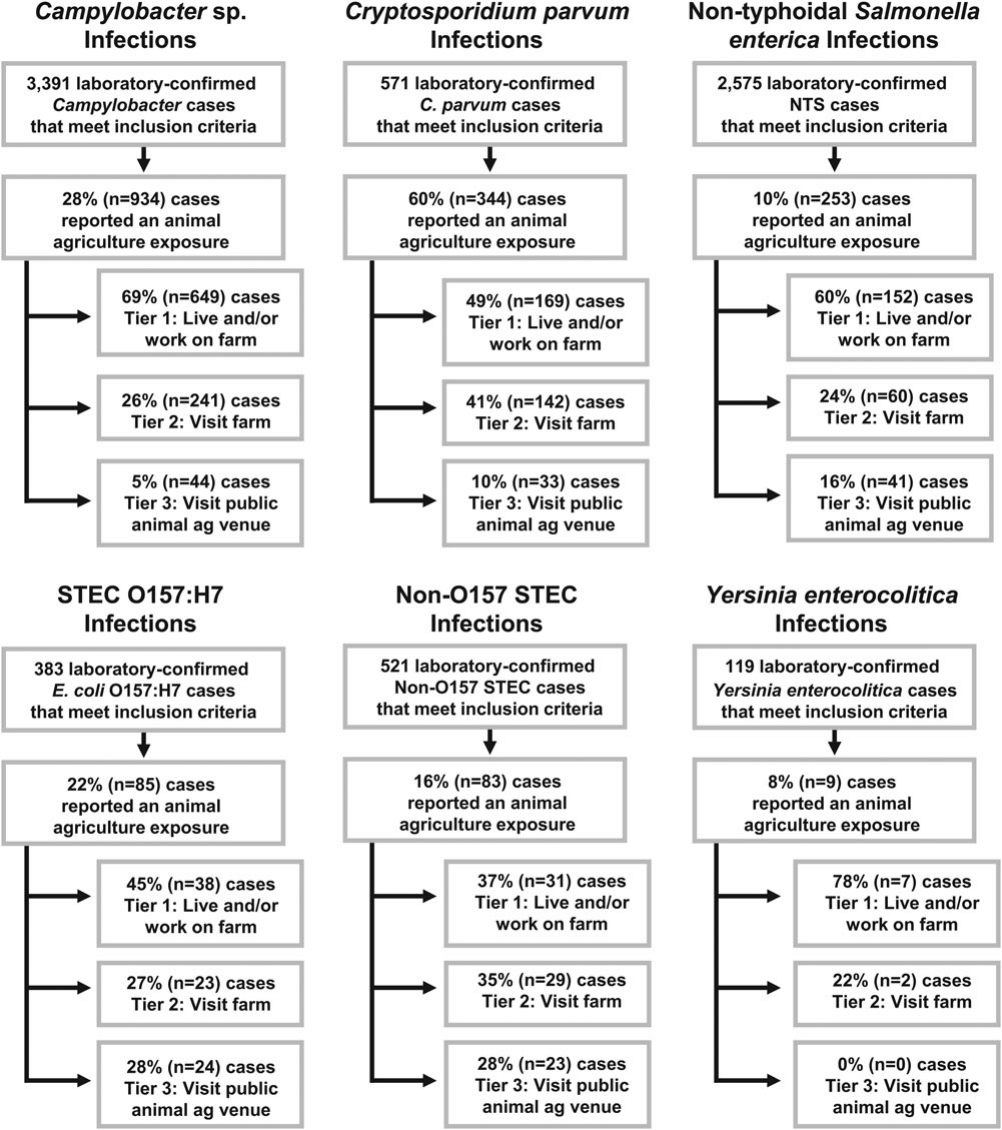
Number and proportion of zoonotic enteric disease cases who reported an animal agriculture exposure, by pathogen and agricultural setting, Minnesota, 2012–2016. NTS = non-typhoidal *S. enterica.* STEC = Shiga toxin-producing *E. coli*.

An animal agriculture exposure was reported by 344 (60%) *C. parvum* cases, 934 (28%) *Campylobacter* cases, 85 (22%) STEC O157 cases, 83 (16%) non-O157 STEC cases, 253 (10%) NTS cases and 9 (8%) *Yersinia enterocolitica* cases (Fig. 2). This included 23 people who had two pathogens detected during the same illness episode, most commonly *Campylobacter*/NTS, *Campylobacter*/*C. parvum* and *C. parvum*/non-O157 STEC. Also included were 12 other people who developed two independent illnesses during the study period, with onset dates separated by at least 4 weeks.

The most common type of animal agriculture exposure reported was living and/or working on a farm (Tier 1), comprising 78% of *Yersinia enterocolitica,* 69% of *Campylobacter*, 60% of NTS, 49% of *C. parvum,* 45% of STEC O157 and 37% of non-O157 STEC cases with an animal agriculture exposure (Fig. 2). Visiting a private farm was the second most commonly reported animal agriculture exposure, except for STEC O157 cases, for whom visiting a public animal agriculture venue was the second most common exposure (28%) (Fig. 2). Because only nine *Yersinia enterocolitica* cases reported an animal agriculture exposure (seven in Tier 1 and two in Tier 2), this pathogen was excluded from further analyses, leaving 1699 cases in the study.

Demographics and clinical features of the 1699 remaining cases who reported an animal agricultural exposure are given in Table 1. Fifty-seven percent were male but gender differed significantly by pathogen. The median age of cases also differed significantly by pathogen. Overall, 43% of cases reported bloody stools and 17% were hospitalized (there were no deaths). Seventeen (10%) STEC cases developed hemolytic uremic syndrome (HUS). Seventy-five percent of cases with cryptosporidiosis experienced weight loss.

**Table 1.** Demographic and clinical characteristics of cases with a laboratory-confirmed zoonotic enteric infection who reported an animal agriculture exposure prior to their illness, by pathogen, Minnesota, 2012–2016, (*n* = 1699)

Characteristic	*Campylobacter* (*n* = 934) No. (%)	*C. parvum* (*n* = 344) No. (%)	NTS^a^ (*n* = 253) No. (%)	STEC^b^ O157 (*n* = 85) No. (%)	Non-O157 STEC^b^ (*n* = 83) No. (%)	Total (*n* = 1699) No. (%)	*P* value^c^
Gender-male	597 (64)	173 (50)	134 (53)	37 (44)	28 (34)	969 (57)	<0.001
Race-white	883 (95)	334 (97)	236 (93)	84 (99)	76 (92)	1613 (95)	0.36
Ethnicity-non-hispanic	909 (97)	336 (98)	242 (96)	84 (99)	80 (96)	1651 (97)	0.20
Median age, year (range)	23 (<1–95)	16 (<1–74)	27 (<1–85)	11 (1–75)	17 (<1–72)	20 (<1–95)	<0.001
Bloody stools^d^	437 (47)	53 (16)	115 (46)	73 (87)	46 (55)	724 (43)	0.19
Hemolytic Uremic Syndrome	NA	NA	NA	15 (18)	2 (3)	17 (10)	NA
Weight loss^d,e^	NA	247 (75)	NA	NA	NA	NA	NA
Median weight loss, lbs^e^ (range)	NA	8 (1–35)	NA	NA	NA	NA	NA
Hospitalization	123 (13)	34 (10)	71 (28)	42 (49)	19 (23)	289 (17)	<0.001
Median length of hospital stay, days (range)	3 (1–40)	3.5 (1–25)	4 (2–29)	4 (2–26)	3 (2–10)	3 (1–40)	0.13
Bacteremia	7 (0.8)	0 (0)	15 (6)	0 (0)	0 (0)	22 (1)	<0.001

a Non-typhoidal *S enterica.*

b Shiga toxin-producing *E. coli.*

c *χ*^2^ used for dichotomous variables and ANOVA used for continuous variables.

d Responses not available for all cases.

e These questions were only asked for *C. parvum* cases.

**Fig. 3.**
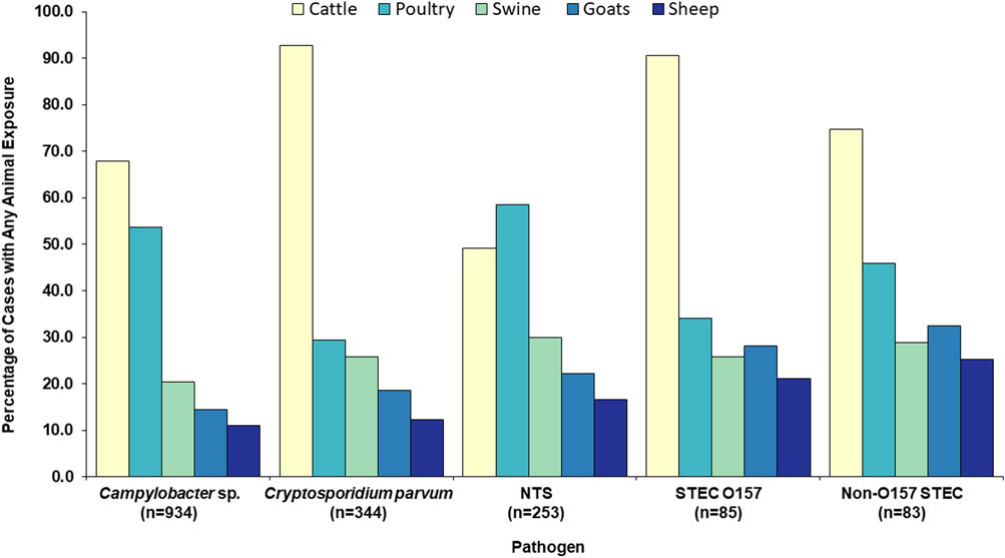
Food production animal types reported by zoonotic enteric disease cases with an animal agriculture exposure, by pathogen, Minnesota, 2012–2016 (*n* = 1699). NTS = non-typhoidal *S. enterica.* STEC = Shiga toxin-producing *E. coli*.

Cattle were the most common animal type in agricultural exposures, reported by 72% of the 1699 cases (Table 2). By pathogen, cattle were the most commonly reported animal type among *Campylobacter, C. parvum,* STEC O157 and non-O157 STEC cases (Table 2, Fig. 3). Among NTS cases, poultry was the most common animal type reported (Table 2, Fig. 3). Seven hundred and forty-eight (44%) cases reported any exposure to more than one animal species.

**Table 2.** Proportion of cases with a laboratory-confirmed zoonotic enteric infection who reported an animal agriculture exposure prior to their illness, by pathogen, type of exposure^a^ and agricultural setting^b^, Minnesota, 2012–2016 (*n* = 1699)

Animal species	*Campylobacter* No. (%)	*C. parvum* No. (%)	NTS^c^ No. (%)	STEC^d^ O157 No. (%)	Non-O157 STEC^d^ No. (%)	Total No. (%)	
Tiers 1, 2 & 3 combined	*n* = 934	*n* = 344	*n* = 253	*n* = 85	*n* = 83	*n* = 1699	*P* value^e^
Cattle-any exposure	633 (68)	319 (93)	124 (49)	77 (91)	62 (75)	1215 (72)	<0.001
Cattle-direct contact	435 (69)	250 (78)	63 (51)	37 (48)	28 (45)	813 (67)	<0.001
Swine-any exposure	191 (20)	89 (26)	76 (30)	22 (26)	24 (29)	402 (24)	0.009
Swine-direct contact	105 (55)	42 (47)	38 (50)	10 (45)	12 (50)	207 (51)	0.74
Poultry-any exposure	501 (54)	101 (29)	148 (58)	29 (34)	38 (46)	817 (48)	<0.001
Poultry-direct contact	350 (70)	54 (53)	77 (52)	13 (45)	17 (45)	511 (63)	<0.001
Goat-any exposure	135 (14)	64 (19)	56 (22)	24 (28)	27 (33)	306 (18)	<0.001
Goat-direct contact	83 (61)	38 (59)	28 (50)	11 (46)	19 (70)	179 (58)	0.25
Sheep-any exposure	103 (11)	42 (12)	42 (17)	18 (21)	21 (25)	226 (13)	<0.001
Sheep-direct contact	56 (54)	21 (50)	18 (43)	8 (44)	13 (62)	116 (51)	0.57
**Tier 1: live and/or work on farm**	*n* = 649	*n* = 169	*n* = 152	*n* = 38	*n* = 31	*n* = 1039	
Cattle-any exposure	440 (68)	155 (92)	70 (46)	35 (92)	20 (65)	720 (69)	<0.001
Cattle-direct contact	349 (79)	137 (88)	44 (63)	20 (57)	12 (60)	562 (78)	<0.001
Swine-any exposure	122 (19)	39 (23)	39 (26)	8 (21)	6 (19)	214 (21)	0.35
Swine-direct contact	88 (72)	25 (64)	25 (64)	4 (50)	5 (83)	147 (69)	0.51
Poultry-any exposure	358 (55)	60 (36)	93 (61)	8 (21)	14 (45)	533 (51)	<0.001
Poultry-direct contact	277 (77)	38 (63)	61 (66)	4 (50)	10 (71)	390 (73)	0.03
Goat-any exposure	79 (12)	28 (17)	13 (9)	5 (13)	8 (26)	133 (13)	0.05
Goat-direct contact	61 (77)	17 (61)	7 (54)	2 (40)	6 (75)	93 (70)	0.12
Sheep-any exposure	63 (10)	13 (8)	16 (11)	1 (3)	3 (10)	96 (9)	0.57
Sheep-direct contact	44 (70)	8 (62)	10 (63)	0 (0)	2 (67)	64 (67)	0.63
**Tier 2: visit private farm**	*n* = 241	*n* = 142	*n* = 60	*n* = 23	*n* = 29	*n* = 495	
Cattle-any exposure	158 (66)	136 (96)	28 (47)	21 (91)	24 (83)	367 (74)	<0.001
Cattle-direct contact	70 (44)	95 (70)	13 (46)	9 (43)	8 (33)	195 (53)	<0.001
Swine-any exposure	44 (18)	27 (19)	16 (27)	1 (4)	3 (10)	91 (18)	0.14
Swine-direct contact	11 (25)	7 (26)	7 (44)	0 (0)	0 (0)	25 (27)	0.43
Poultry-any exposure	117 (49)	27 (19)	35 (58)	8 (35)	14 (48)	201 (41)	<0.001
Poultry-direct contact	65 (56)	11 (41)	10 (29)	5 (63)	4 (29)	95 (47)	0.02
Goat-any exposure	25 (10)	14 (10)	8 (13)	4 (17)	4 (14)	55 (11)	0.67
Goat-direct contact	7 (28)	5 (36)	3 (38)	1 (25)	3 (75)	19 (35)	0.47
Sheep-any exposure	14 (6)	10 (7)	5 (8)	1 (4)	2 (7)	32 (6)	0.91
Sheep-direct contact	4 (29)	4 (40)	3 (60)	1 (100)	1 (50)	13 (41)	0.53
**Tier 3: visit public animal agriculture venue**	*n* = 44	*n* = 33	*n* = 41	*n* = 24	*n* = 23	*n* = 165	
Cattle-any exposure	35 (80)	28 (85)	26 (63)	21 (88)	18 (78)	128 (78)	0.16
Cattle-direct contact	16 (46)	18 (64)	6 (23)	8 (38)	8 (44)	56 (44)	0.05
Swine-any exposure	25 (57)	23 (70)	21 (51)	13 (54)	15 (65)	97 (59)	0.51
Swine-direct contact	6 (24)	10 (43)	6 (29)	6 (46)	7 (47)	35 (36)	0.41
Poultry-any exposure	26 (59)	14 (42)	20 (49)	13 (54)	10 (43)	83 (50)	0.59
Poultry-direct contact	8 (31)	5 (36)	6 (30)	4 (31)	3 (30)	26 (31)	1.00
Goat-any exposure	31 (70)	22 (67)	35 (85)	15 (63)	15 (65)	118 (72)	0.23
Goat-direct contact	15 (48)	16 (73)	18 (51)	8 (53)	10 (67)	67 (57)	0.38
Sheep-any exposure	26 (59)	19 (58)	21 (51)	16 (67)	16 (70)	98 (59)	0.61
Sheep-direct contact	8 (31)	9 (47)	5 (24)	7 (44)	10 (63)	39 (40)	0.14

a Any exposure = direct contact or indirect contact (i.e. contact with an animal environment). Direct contact data given as a subset of any exposure for each animal type.

b Agricultural setting defined as: Tier 1 – living and/or working on a farm; Tier 2 – visiting a private farm and Tier 3 – visiting a public animal agriculture venue.

c Non-typhoidal *S. enterica.*

d Shiga toxin-producing *E. coli.*

e *χ*^2^ used for dichotomous variables and ANOVA used for continuous variables.

**Fig. 4.**
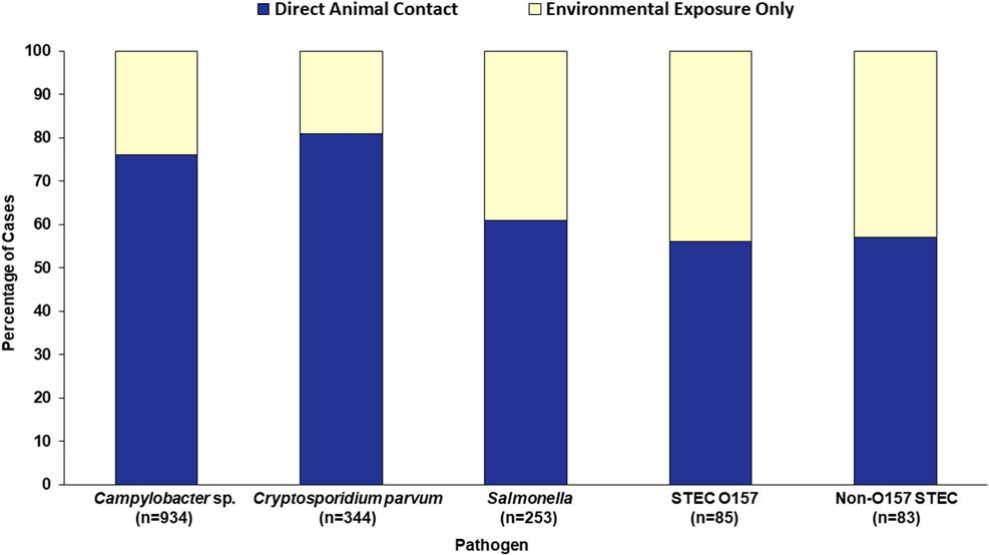
Proportion of cases with an animal agriculture exposure who reported direct animal contact *vs.* environmental exposure only, by pathogen, Minnesota, 2012–2016 (*n* = 1699). NTS = non-typhoidal *S. enterica.* STEC = Shiga toxin-producing *E. coli.*

Seventy-three percent (*n* = 1240) of the 1699 cases with an animal agriculture exposure reported direct animal contact, whereas 27% (*n* = 459) reported exposure only to animal environments (Fig. 4). Of those with direct animal contact, 399 (32%) had contact with more than one species. Raw milk consumption was reported by 140 (8%) of the 1699 cases, including 107 *Campylobacter*, 19 *C. parvum,* 6 *Salmonella,* 6 non-O157 STEC and 2 STEC O157 cases.

### Living and/or working on a farm (Tier 1)

#### By pathogen

The estimated cumulative incidence of zoonotic enteric infections for people living and/or working on a farm with food production animals per 10 000 population during 2012–2016 compared to other Minnesotans was as follows: all pathogens combined, 147 *vs.* 18.5 (7.9 times greater, *P* < 0.001); *Campylobacter*, 92.0 *vs.* 7.5 (*P* < 0.001); *C. parvum,* 23.8 *vs.* 1.2 (*P* < 0.001); NTS, 21.4 *vs.* 7.4 (*P* < 0.001); STEC O157, 5.4 *vs.* 1.1 (*P* < 0.001) and non-O157 STEC, 4.4 *vs.* 1.3 (*P* < 0.001). In the sensitivity analysis in which workers, operators and children <13 years on crop farms were added to the initial denominator, the estimated cumulative incidence of zoonotic enteric infections for people living and/or working on a farm was 42.7 per 10 000 population *vs.* 19.1 for other Minnesotans (2.2 times greater, *P* < 0.001).

Of the 1039 cases who lived and/or worked on a farm (Tier 1), 62% had *Campylobacter* infections, 16% had *C. parvum* infections, 15% had NTS infections, 4% had STEC O157 infections and 3% had non-O157 STEC infections (Table 2). More than two-thirds of Tier 1 cases reported any exposure to cattle, with a majority of those reporting direct contact; however, these proportions differed by pathogen (Table 2). Seven hundred ninety-six (77%) of Tier 1 cases also completed the more detailed agricultural exposure questionnaire. Of these, 59% of cases reported living and/or working on a full-time farm *vs.* a part-time farm. Tier 1 cases reported living and/or working on the farm for a median of 4 years prior to their illness, but this varied significantly by pathogen (Table 3). A majority of cases missed school/work, and this varied by pathogen as well (Table 3). Twenty percent of Tier 1 cases reported ill animals prior to their illness, including 40% of *C. parvum* cases (Table 3).

**Table 3.** Characteristics, knowledge, attitudes and practices (KAP) reported among laboratory-confirmed zoonotic enteric disease cases who lived and/or worked on a farm with food production animals prior to their illness, Minnesota, 2012–2016 (*n* = 797)^a,b^

Characteristics and KAP	*Campylobacter* No. (%)	*C. parvum* No. (%)	NTS^c^ No. (%)	STEC^d^ O157 No. (%)	Non-O157 STEC^d^ No. (%)	Total No. (%)	*P* value^e^
Tier 1: live and/or work on a farm	*n* = 503	*n* = 133	*n* = 104	*n* = 30	*n* = 26	*n* = 796	
Fulltime farm	307 (61)	77 (58)	57 (55)	18 (60)	13 (50)	472 (59)	0.17
Median time on farm, years (range)	4 (0.01–74)	2 (0.08–74)	9 (0.08–69)	4 (0.08–48)	3 (0.17–50)	4 (0.01–74)	<0.001
Previous history of live/work on farm	192 (40)	35 (28)	43 (42)	8 (27)	5 (19)	283 (36)	0.60
Missed school or work	314 (66)	85 (67)	57 (56)	15 (50)	13 (50)	484 (63)	0.02
Animals ill prior to case illness	86 (18)	50 (40)	17 (17)	2 (7)	8 (32)	163 (20)	0.78
Awareness of zoonoses	426 (87)	101 (81)	93 (93)	28 (93)	24 (96)	672 (88)	0.07
Measures exist to reduce risk	179 (42)	49 (49)	33 (35)	11 (39)	7 (29)	279 (42)	0.14
Change in practices	172 (36)	53 (44)	32 (32)	10 (33)	10 (42)	277 (37)	0.66
Requested educational materials	258 (53)	74 (60)	65 (65)	21 (70)	16 (64)	434 (57)	0.005

a Includes cases who completed an additional detailed interview regarding farm characteristics, exposures and prevention measures.

b Responses not available for all cases for some variables.

c Non-typhoidal *S. enterica.*

d Shiga toxin-producing *E. coli.*

e *χ*^2^ used for dichotomous variables and ANOVA used for continuous variables.

A large majority of Tier 1 cases understood the concept of zoonoses (Table 3). However, only 42% of cases reported there were safety measures in place to reduce the risk of zoonoses on the farm, and 37% reported a change in their practices after their illness (Table 3).

#### By food animal type

**Fig. 5.**
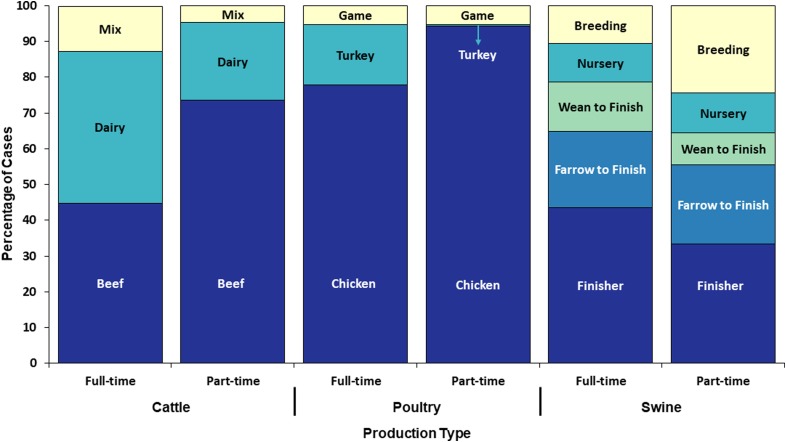
Proportion of cases with an animal agriculture exposure who lived and/or worked on a full-time farm *vs.* a part-time farm by food animal category and production type, Minnesota, 2012–2016 (*n* = 544).

Seven hundred and twenty (69%) of the 1039 Tier 1 cases reported exposure to cattle; of these, 61% were infected with *Campylobacter*, 22% with *C. parvum*, 10% with NTS, 5% with STEC O157 and 3% with non-O157 STEC (Table 4). Five hundred and forty-four (76%) of the 720 cases completed the Tier 1-specific questionnaire; of these, 69% reported living and/or working on a full-time operation, and 31% reported living and/or working on a part-time farm (Fig. 5).

**Table 4.** Food animal types reported by people who live and/or work on a farm with food production animals (Tier 1) with laboratory-confirmed zoonotic enteric infections and animal agriculture exposure^a^, by pathogen, Minnesota, 2012–2016 (*n* = 1039)

Pathogen	Cattle exposure (*n* = 720) No. (%)	Poultry exposure (*n* = 533) No. (%)	Swine exposure (*n* = 214) No. (%)	Goat exposure (*n* = 133) No. (%)	Sheep exposure (*n* = 96) No. (%)
*Campylobacter*	440 (61)	358 (67)	122 (57)	79 (59)	63 (66)
*C. parvum*	155 (22)	60 (11)	39 (18)	28 (21)	13 (14)
NTS^b^	70 (10)	93 (17)	39 (18)	13 (10)	16 (17)
STEC^c^ O157	35 (5)	8 (2)	8 (4)	5 (4)	1 (1)
Non-O157 STEC^c^	20 (3)	14 (3)	6 (3)	8 (6)	3 (3)

a Exposure = direct contact *or indirect contact (i.e. contact with an animal environment).*

b Non-typhoidal *S. enterica.*

c Shiga toxin-producing *E. coli.*

Five hundred and thirty-three (51%) of the 1039 Tier 1 cases reported poultry exposure; among these, 67% were infected with *Campylobacter* and 17% with NTS (Table 4). Four hundred and twenty-three (79%) of 533 cases completed the Tier 1-specific questionnaire. One hundred and ninety-three (46%) reported living and/or working on a full-time farm; of these, 77% cases reported exposures to poultry on full-time poultry farms and 23% of cases reported exposure to hobby poultry kept on full-time livestock farms (Fig. 5). Of the 230 (54%) cases on a part-time farm, 70% reported having other food animals in addition to chickens.

Two hundred and fourteen (21%) of the 1039 Tier 1 cases reported swine exposure; of these, 57% were infected with *Campylobacter*, and 18% with NTS (Table 4). One hundred and seventy-six (82%) cases completed the Tier 1-specific questionnaire; of these, 111 (63%) cases reported living and/or working on a full-time farm and 65 (37%) reported a part-time farm (Fig. 5).

### Visiting a private farm (Tier 2)

Among the 495 cases who visited a private farm (Tier 2), 49% had *Campylobacter*, 29% *C. parvum*, 12% NTS, 6% non-O157 STEC and 5% STEC O157 infections (Table 2). Cattle were the most commonly reported animal type for all pathogens except NTS, for which 58% of cases reported poultry exposure (Table 2). STEC O157, *C. parvum*, *Campylobacter* and non-O157 STEC cases were significantly more likely to report cattle exposure than one or more of the other pathogens (*P* = 0.02 to <0.001; data in Table 2). *Campylobacter* and NTS cases were significantly more likely to report poultry exposure than *C. parvum* cases (both *P* < 0.001) (data in Table 2).

### Visiting a public animal agriculture venue (Tier 3)

Among the 165 cases who visited a public animal agriculture venue (Tier 3), 27% had *Campylobacter,* 25% NTS, 20% *C. parvum,* 15% STEC O157 and 14% non-O157 STEC infections (Table 2). Seventy-eight percent of Tier 3 cases reported any exposure to cattle and 44% reported direct cattle contact. Seventy-eight percent of Tier 3 cases reported any exposure to goats, and 57% reported direct goat contact, making goats the most frequently contacted animal in these settings (Table 2).

### Outbreak analyses

During 2012–2016, 126 enteric disease cases in Minnesota occurred as part of 23 outbreaks associated with animal agriculture settings, compared to the 1699 sporadic cases with exposure to an animal agriculture setting. Therefore, outbreak-associated cases made up only 7% of the total number of cases overall (0.2% to 29% depending on the pathogen) with an animal agriculture exposure (Table 5).

**Table 5.** Sporadic zoonotic enteric disease cases reporting an animal agriculture exposure, outbreak cases associated with an animal agriculture outbreak setting and outbreak cases associated with other transmission routes, by pathogen, Minnesota, 2012–2016

Type of case	Campylobacter No.	*C. parvum* No.	NTS^b^ No.	STEC^c^ O157 No.	Non-O157 STEC^c^ No.	Total No.
Sporadic, animal ag exposure	934	344	253	85	83	1699
Outbreak, animal ag setting	2	34	55	34	1	126
Outbreak, all other transmission types^a^	53	80	458	135	59	785

a Foodborne, person-to-person, waterborne, contact with non-agricultural animals and undetermined.

b Non-typhoidal *S. enterica.*

c Shiga toxin-producing *E. coli.*

When considering all enteric disease outbreak-associated cases from all transmission routes during 2012–2016, 14% were associated with an animal agriculture setting while the remaining 86% were associated with other transmission routes (foodborne, person-to-person, waterborne, undetermined and contact with non-agricultural animals). Comparing outbreak-associated cases by pathogen, 30% of *C. parvum*, 20% of STEC O157, 11% of NTS, 4% of *Campylobacter* and 2% of non-O157 STEC cases were part of outbreaks associated with an animal agriculture setting (Table 5).

## Discussion

Our 5-year, population-based study provides strong evidence that animal agriculture exposures account for a higher proportion of sporadic, domestically acquired, enteric zoonotic pathogen cases than previously appreciated, and the burden of enteric zoonoses among agricultural workers is much higher than in the general population.

Twenty-three percent of zoonotic, domestically acquired, sporadic, enteric disease cases reported to MDH during 2012–2016 reported an animal agriculture exposure during their incubation period. Among individual pathogens, the proportion of cases with an animal agriculture exposure was the highest for *C. parvum* cases, at a striking rate of 60%. The proportions for other pathogens ranged from 8% for *Y. enterocolitica* to 28% for *Campylobacter* cases; these proportions were also generally higher than most previous estimates [1, 15, 25, 26]. Hale *et al*. estimated the proportion of illnesses in the United States attributable to contact with animals of all types, not just food animals but also companion animals, based on case-control studies and outbreak data [1]. Therefore, comparisons of our findings to estimates from the Hale study are not straightforward. Yet, in our study, the proportion of cases that could be explained by agricultural animal exposures alone greatly exceeded the upper end of the range of the Hale estimates for *Cryptosporidium*, STEC O157 and *Y. enterocolitica*. Our proportions were at the top end of the Hale ranges for *Campylobacter* and non-O157 STEC and they were similar to the Hale estimate for *Salmonella* [1].

In terms of absolute numbers of cases, *Campylobacter* was the most important zoonotic pathogen in agricultural workers, their families and others with animal agriculture exposures. *C*. *parvum* and NTS also caused large numbers of illnesses in these populations. STEC O157 and non-O157 STEC combined caused 10% of the illnesses among those with animal agriculture exposures, resulting in 17 HUS cases. Eleven of the 17 HUS cases occurred in people who lived on a farm (*n* = 5) or visited a private farm (*n* = 6), and six HUS cases occurred in visitors to public animal contact venues. In addition to the well-publicized risk associated with public venues, HUS is also an important concern for private farm residents and visitors.

Our study considered laboratory-confirmed cases only. The actual number of cases with animal agriculture exposures is likely much higher. In the United States, multiplier estimates for the number of cases that occur for each laboratory-confirmed case detected range from 26 for STEC O157 to over 100 for non-O157 STEC and *Y. enterocolitica* [27]. The number of *C. parvum* cases is further underestimated because 33% of *Cryptosporidium* specimens submitted to MDH during the study period could not be identified to species, and thus were not included in this study.

Living and/or working on a farm was the most frequent type of animal agriculture exposure reported (61%). This is consistent with findings of a study in South Dakota, another heavily agricultural state [28]. In Minnesota, visiting a private farm with food production animals was also a common exposure, accounting for 29% of animal agriculture exposures. Thus, the vast majority of people who acquire an enteric zoonosis from food animal contact do so through living on, working on, or visiting a private farm.

Visiting a public animal contact venue accounted for 10% of animal agriculture exposures. While this was the lowest percentage of all three tiers, the 165 sporadic cases with this exposure were greater than the 126 cases associated with outbreaks at Minnesota public animal agriculture venues during the study period. Also, these venues were the second most common exposure setting for STEC O157 cases which is of concern because of the increased risk of HUS in young children, a primary target audience for public animal contact venues.

The NORA Agriculture, Forestry and Fishing Sector include agricultural workers and their families as an occupational category. We demonstrated that the incidence of enteric zoonoses among people who live and/or work on farms with food production animals in Minnesota is much higher than in the general population 8 times higher overall, and up to 20 times higher depending on the pathogen. This underscores the importance of enteric zoonoses as an occupational hazard in animal agriculture.

Cattle were by far the most commonly reported animal type among cases infected with *C*. *parvum*, *Campylobacter*, STEC O157 and non-O157 STEC. Statistically significant associations with cattle exposure were identified for cases infected with *C. parvum*, *Campylobacter* and STEC O157. Poultry, primarily chickens, was the most commonly reported animal type among *Salmonella* cases, and a distinct second for *Campylobacter* cases; statistically significant associations with poultry exposures were identified for both pathogens. Swine were the third most commonly reported animal type for both *Campylobacter* and *Salmonella* cases. Goats were the most commonly reported species with which cases (all pathogens) who visited public animal contact venues had direct contact, and likely represent an important source of STEC exposure for visitors to these settings. These findings are consistent with the known reservoirs of our study pathogens.

In Minnesota during the study period, 14% of all enteric outbreak cases were associated with animal contact in agriculture settings (i.e. *vs.* foodborne and other transmission routes) (Table 5). During the same period, the number of sporadic illness cases reporting animal agriculture exposures was 13.5 times greater than the number of cases that were part of outbreaks associated with animal agriculture settings. This suggests the burden of illness associated with animal agricultural settings is far greater than indicated by recognized outbreaks.

This study had several limitations. First, because the cases were sporadic, a cause and effect relationship between the animal agriculture exposure and the case could not be confirmed. However, zoonotic disease outbreak investigations and case-control studies have consistently demonstrated associations with agricultural exposures [1–8]. While exposure to farm animal environments is a well-documented means of transmission [8–10, 29–31], the finding that 73% of cases reported direct animal contact increases the likelihood that the animal agriculture exposure represented the true exposure for most cases. Other exposures (e.g. raw milk, other foods, well water) could have been the actual source of illness for at least some cases; however, many of these other possible exposures are also of specific concern to agricultural populations.

Another limitation is that our findings are not generalizable across the United States, as the importance and types of animal agriculture vary dramatically by region. States with a greater agricultural economic base than Minnesota, such as South Dakota [28], can expect to have an even higher burden of illness associated with animal agriculture. Conversely, that burden is likely to be less in states in which animal agriculture is not as economically important.

Finally, deriving an estimate of the number of people who live and/or work on farms with food production animals in Minnesota, to establish a denominator for cumulative incidence calculations, was difficult. For example, people who live and/or work on a farm on which 51% of the income is derived through crop production and 49% through livestock/poultry production would not be counted as animal agriculture workers by the Agriculture Census [21, 22]. Therefore, the denominator used in our base incidence calculation was almost certainly an underestimate. To address this, we also conducted a sensitivity analysis in which we added all Minnesota crop farm workers and operators, and children <13 years who live on crop farms, to the base denominator to provide a more conservative incidence estimate. In both instances, the incidence of zoonotic enteric infections among those living and/or working on farms over the 5-year study period was significantly higher than in other Minnesotans (almost 8 times higher in the first estimate and 2.2 times higher in the second estimate).

## Conclusions

The burden of enteric zoonoses among agricultural workers and others with animal agriculture exposures appears to be far greater than previously appreciated. Disease prevention efforts have been targeted primarily at public animal contact venues; however, living on, working on or even visiting a private farm are likely much more frequent sources of disease. Zoonotic enteric disease is an under-recognized risk associated with farming. Targeted outreach to raise awareness among agricultural workers and their families is urgently needed. However, detailed, pathogen-specific analyses are necessary to further understand the problem, including specific populations at risk, food animal production settings and risk factors for infection. Only then can appropriate prevention measures be envisioned, implemented and evaluated.

## Supplementary material

For supplementary material accompanying this paper visit https://doi.org/10.1017/S0950268819002309. click here to view supplementary material
