# Supplementary material for: Animal agriculture exposures among Minnesota residents with zoonotic enteric infections, 2012–2016
Source: Epidemiol Infect. 2020 Mar 16;148:e55. doi: 10.1017/S0950268819002309 (PMC7078579; doi:10.1017/S0950268819002309)
Supplement: Supplementary file 1 [file S0950268819002309sup001.zip › Klumb Supplementary Figure S4.docx]

*Epidemiology and Infection*

Animal Agriculture Exposures among Minnesota Residents with Zoonotic Enteric Infections, 2012-2016

C. A. Klumb, J. M. Scheftel, K. E. Smith

Supplementary Material

**Figure S4: Tier 3 questionnaire – Visit a public animal contact venue**

**UMASH Interview**

**Tier 3: Cases who visited a public animal contact venue**

Patient’s Name (last, first): ________________________________________ DOB: ___/___/___

Parent’s Name (if child): __________________________________________ Onset: ___/___/___

Illness: ____________________________________ Phone number: __________________________

1. What is the name of the venue you went to?_____________________________________________
2. What county was venue located in?____________________________­­­­­­­________________________
3. What dates did you/your child visit the venue in the 7(14) days prior to illness? ______________________________
4. I am going to ask you about the different animals that may have been at the venue and activities you did. *(Interviewer: Please check the appropriate box or fill in the number for each animal in the table below.)*

|  | Animal Present?  (Enviro Contact) | Direct Contact? | Feed Animals? | Enter Pen? |
| --- | --- | --- | --- | --- |
|  | Yes No DK | Yes No DK | Yes No DK | Yes No DK |
| Cows | 🞏 🞏 🞏 | 🞏 🞏 🞏 | 🞏 🞏 🞏 | 🞏 🞏 🞏 |
| Calves | 🞏 🞏 🞏 | 🞏 🞏 🞏 | 🞏 🞏 🞏 | 🞏 🞏 🞏 |
| Pigs | 🞏 🞏 🞏 | 🞏 🞏 🞏 | 🞏 🞏 🞏 | 🞏 🞏 🞏 |
| Piglets | 🞏 🞏 🞏 | 🞏 🞏 🞏 | 🞏 🞏 🞏 | 🞏 🞏 🞏 |
| Sheep | 🞏 🞏 🞏 | 🞏 🞏 🞏 | 🞏 🞏 🞏 | 🞏 🞏 🞏 |
| Lambs | 🞏 🞏 🞏 | 🞏 🞏 🞏 | 🞏 🞏 🞏 | 🞏 🞏 🞏 |
| Goats | 🞏 🞏 🞏 | 🞏 🞏 🞏 | 🞏 🞏 🞏 | 🞏 🞏 🞏 |
| Kids | 🞏 🞏 🞏 | 🞏 🞏 🞏 | 🞏 🞏 🞏 | 🞏 🞏 🞏 |
| Chickens | 🞏 🞏 🞏 | 🞏 🞏 🞏 | 🞏 🞏 🞏 | 🞏 🞏 🞏 |
| Chicks | 🞏 🞏 🞏 | 🞏 🞏 🞏 | 🞏 🞏 🞏 | 🞏 🞏 🞏 |
| Turkeys | 🞏 🞏 🞏 | 🞏 🞏 🞏 | 🞏 🞏 🞏 | 🞏 🞏 🞏 |
| Ducks | 🞏 🞏 🞏 | 🞏 🞏 🞏 | 🞏 🞏 🞏 | 🞏 🞏 🞏 |
| Ducklings | 🞏 🞏 🞏 | 🞏 🞏 🞏 | 🞏 🞏 🞏 | 🞏 🞏 🞏 |
| Horse/donkey/mule | 🞏 🞏 🞏 | 🞏 🞏 🞏 | 🞏 🞏 🞏 | 🞏 🞏 🞏 |
| Llama/alpaca | 🞏 🞏 🞏 | 🞏 🞏 🞏 | 🞏 🞏 🞏 | 🞏 🞏 🞏 |
| Deer/elk | 🞏 🞏 🞏 | 🞏 🞏 🞏 | 🞏 🞏 🞏 | 🞏 🞏 🞏 |
| Cats | 🞏 🞏 🞏 | 🞏 🞏 🞏 | 🞏 🞏 🞏 | 🞏 🞏 🞏 |
| Kittens | 🞏 🞏 🞏 | 🞏 🞏 🞏 | 🞏 🞏 🞏 | 🞏 🞏 🞏 |
| Dog | 🞏 🞏 🞏 | 🞏 🞏 🞏 | 🞏 🞏 🞏 | 🞏 🞏 🞏 |
| Puppies | 🞏 🞏 🞏 | 🞏 🞏 🞏 | 🞏 🞏 🞏 | 🞏 🞏 🞏 |
| Other:__________ | 🞏 🞏 🞏 | 🞏 🞏 🞏 | 🞏 🞏 🞏 | 🞏 🞏 🞏 |

1. Were there sinks with soap available for hand washing? 🞏 Yes 🞏 No 🞏 DK

***If yes,*** did you/your child wash your hands after contact with the

animals or their environment? 🞏 Yes 🞏 No 🞏 DK

***If yes,*** did you/your child use soap? 🞏 Yes 🞏 No 🞏 DK

1. Did you have paper/cloth towels to dry your hands with after you washed them? 🞏 Yes 🞏 No 🞏 DK

***If yes,*** did you use it? 🞏 Yes 🞏 No 🞏 DK

1. Was hand sanitizer available? 🞏 Yes 🞏 No 🞏 DK

***If yes,*** did you/your child use it after contact with the animals

or their environment? 🞏 Yes 🞏 No 🞏 DK

1. Did you/your child eat, drink, chew, or smoke anything while with the animals? 🞏 Yes 🞏 No 🞏 DK

1. Were any signs posted reminding you to wash your hands or to keep food and 🞏 Yes 🞏 No 🞏 DK

beverages out of the animal areas?

1. Did you/your child do any other activities at the venue

(e.g., apple picking, rides)? 🞏 Yes 🞏 No 🞏 DK

***If yes,*** what activities?_________________________________________________

1. Did you/your child remove your shoes once you got home? 🞏 Yes 🞏 No 🞏 DK
2. Did you/your child change your clothes once you got home? 🞏 Yes 🞏 No 🞏 DK
3. Would you be interested in receiving educational materials about farm animals and your illness?

🞏 Yes 🞏 No

🞏 Email: _____________________________________

🞏 Home address *(confirm address from CRF)*

*That concludes our interview, thank you for speaking with me. Do you have any questions?*

Comments: ­_______________________________________________________________________

_________________________________________________________________________________
