# Supplementary material for: Animal agriculture exposures among Minnesota residents with zoonotic enteric infections, 2012–2016
Source: Epidemiol Infect. 2020 Mar 16;148:e55. doi: 10.1017/S0950268819002309 (PMC7078579; doi:10.1017/S0950268819002309)
Supplement: Supplementary file 1 [file S0950268819002309sup001.zip › Klumb Supplementary Figure S3.docx]

*Epidemiology and Infection*

Animal Agriculture Exposures among Minnesota Residents with Zoonotic Enteric Infections, 2012-2016

C. A. Klumb, J. M. Scheftel, K. E. Smith

Supplementary Material

**Figure S3: Tier 2 questionnaire – Visit a private farm**

**UMASH Interview**

**Tier 2: Cases who visited a private farm or farms**

Patient’s Name (last, first): ________________________________________ DOB: ___/____/___

Parent’s Name (if child): __________________________________________ Onset: ___/___/___

Illness:_______________________ Phone Number: ­­­­­­­­­_________________________________

1. What county is the farm(s) located in? ________________________________________
2. What dates did you/your child visit the venue in the 7(14) days prior to illness? _____________________________
3. How often do you/your child visit the farm(s)?

🞏 1-2x only 🞏 Daily 🞏 Weekly 🞏 Monthly

🞏 Quarterly 🞏 Yearly 🞏 Unknown

1. I am going to ask you about the different animals that may have been on the farm & the activities you did. *(Interviewer: Please check the appropriate box or fill in the number for each animal in the table below.)*

|  | Animal Present?  (Enviro Contact) | Direct Contact? | Feed Animals? | Chores? | Describe  Chores | Enter Pen? | Other activities with animals? | Describe Other Activities | Head Count |
| --- | --- | --- | --- | --- | --- | --- | --- | --- | --- |
| Cows | 🞏 Yes  🞏 No  🞏 DK | 🞏 Yes  🞏 No  🞏 DK | 🞏 Yes  🞏 No  🞏 DK | 🞏 Yes  🞏 No  🞏 DK |  | 🞏 Yes  🞏 No  🞏 DK | 🞏 Yes  🞏 No  🞏 DK |  |  |
| Calves | 🞏 Yes  🞏 No  🞏 DK | 🞏 Yes  🞏 No  🞏 DK | 🞏 Yes  🞏 No  🞏 DK | 🞏 Yes  🞏 No  🞏 DK |  | 🞏 Yes  🞏 No  🞏 DK | 🞏 Yes  🞏 No  🞏 DK |  |  |
| Pigs | 🞏 Yes  🞏 No  🞏 DK | 🞏 Yes  🞏 No  🞏 DK | 🞏 Yes  🞏 No  🞏 DK | 🞏 Yes  🞏 No  🞏 DK |  | 🞏 Yes  🞏 No  🞏 DK | 🞏 Yes  🞏 No  🞏 DK |  |  |
| Piglets | 🞏 Yes  🞏 No  🞏 DK | 🞏 Yes  🞏 No  🞏 DK | 🞏 Yes  🞏 No  🞏 DK | 🞏 Yes  🞏 No  🞏 DK |  | 🞏 Yes  🞏 No  🞏 DK | 🞏 Yes  🞏 No  🞏 DK |  |  |
| Sheep | 🞏 Yes  🞏 No  🞏 DK | 🞏 Yes  🞏 No  🞏 DK | 🞏 Yes  🞏 No  🞏 DK | 🞏 Yes  🞏 No  🞏 DK |  | 🞏 Yes  🞏 No  🞏 DK | 🞏 Yes  🞏 No  🞏 DK |  |  |
| Lambs | 🞏 Yes  🞏 No  🞏 DK | 🞏 Yes  🞏 No  🞏 DK | 🞏 Yes  🞏 No  🞏 DK | 🞏 Yes  🞏 No  🞏 DK |  | 🞏 Yes  🞏 No  🞏 DK | 🞏 Yes  🞏 No  🞏 DK |  |  |
| Goats | 🞏 Yes  🞏 No  🞏 DK | 🞏 Yes  🞏 No  🞏 DK | 🞏 Yes  🞏 No  🞏 DK | 🞏 Yes  🞏 No  🞏 DK |  | 🞏 Yes  🞏 No  🞏 DK | 🞏 Yes  🞏 No  🞏 DK |  |  |
| Kids | 🞏 Yes  🞏 No  🞏 DK | 🞏 Yes  🞏 No  🞏 DK | 🞏 Yes  🞏 No  🞏 DK | 🞏 Yes  🞏 No  🞏 DK |  | 🞏 Yes  🞏 No  🞏 DK | 🞏 Yes  🞏 No  🞏 DK |  |  |
| Chickens | 🞏 Yes  🞏 No  🞏 DK | 🞏 Yes  🞏 No  🞏 DK | 🞏 Yes  🞏 No  🞏 DK | 🞏 Yes  🞏 No  🞏 DK |  | 🞏 Yes  🞏 No  🞏 DK | 🞏 Yes  🞏 No  🞏 DK |  |  |
| Chicks | 🞏 Yes  🞏 No  🞏 DK | 🞏 Yes  🞏 No  🞏 DK | 🞏 Yes  🞏 No  🞏 DK | 🞏 Yes  🞏 No  🞏 DK |  | 🞏 Yes  🞏 No  🞏 DK | 🞏 Yes  🞏 No  🞏 DK |  |  |
| Turkeys | 🞏 Yes  🞏 No  🞏 DK | 🞏 Yes  🞏 No  🞏 DK | 🞏 Yes  🞏 No  🞏 DK | 🞏 Yes  🞏 No  🞏 DK |  | 🞏 Yes  🞏 No  🞏 DK | 🞏 Yes  🞏 No  🞏 DK |  |  |
| Ducks | 🞏 Yes  🞏 No  🞏 DK | 🞏 Yes  🞏 No  🞏 DK | 🞏 Yes  🞏 No  🞏 DK | 🞏 Yes  🞏 No  🞏 DK |  | 🞏 Yes  🞏 No  🞏 DK | 🞏 Yes  🞏 No  🞏 DK |  |  |
| Ducklings | 🞏 Yes  🞏 No  🞏 DK | 🞏 Yes  🞏 No  🞏 DK | 🞏 Yes  🞏 No  🞏 DK | 🞏 Yes  🞏 No  🞏 DK |  | 🞏 Yes  🞏 No  🞏 DK | 🞏 Yes  🞏 No  🞏 DK |  |  |
| Horse/mule/donkey | 🞏 Yes  🞏 No  🞏 DK | 🞏 Yes  🞏 No  🞏 DK | 🞏 Yes  🞏 No  🞏 DK | 🞏 Yes  🞏 No  🞏 DK |  | 🞏 Yes  🞏 No  🞏 DK | 🞏 Yes  🞏 No  🞏 DK |  |  |
| Llama/alpaca | 🞏 Yes  🞏 No  🞏 DK | 🞏 Yes  🞏 No  🞏 DK | 🞏 Yes  🞏 No  🞏 DK | 🞏 Yes  🞏 No  🞏 DK |  | 🞏 Yes  🞏 No  🞏 DK | 🞏 Yes  🞏 No  🞏 DK |  |  |
| Deer/elk | 🞏 Yes  🞏 No  🞏 DK | 🞏 Yes  🞏 No  🞏 DK | 🞏 Yes  🞏 No  🞏 DK | 🞏 Yes  🞏 No  🞏 DK |  | 🞏 Yes  🞏 No  🞏 DK | 🞏 Yes  🞏 No  🞏 DK |  |  |
| Other:_______________ | 🞏 Yes  🞏 No  🞏 DK | 🞏 Yes  🞏 No  🞏 DK | 🞏 Yes  🞏 No  🞏 DK | 🞏 Yes  🞏 No  🞏 DK |  | 🞏 Yes  🞏 No  🞏 DK | 🞏 Yes  🞏 No  🞏 DK |  |  |

1. *Interviewer: Internal use only. Please check “yes” or “no” based on answers above.*

Did you/your child have contact with any of the animals’ environments? 🞏 Yes 🞏 No

1. Did you/your child wear any personal protective equipment while with the animals? ***If yes,*** check all that apply.

🞏 Yes 🞏 No 🞏 DK 🞏 NA

🞏 Gloves 🞏 Boots 🞏 Work clothes/coveralls 🞏 Facial Protection (Mask/glasses)

1. In the 7 (14) days prior to illness did you/your child come into contact with manure in the house via dirty work clothes or boots? 🞏 Yes 🞏 No 🞏DK
2. Were there sinks with soap available for hand washing? 🞏 Yes 🞏 No 🞏 DK

***If yes,*** did you/your child wash your hands after contact with the

animals or their environment? 🞏 Yes 🞏 No 🞏 DK

***If yes,*** did you/your child use soap? 🞏 Yes 🞏 No 🞏 DK

1. Did you have paper/cloth towels to dry your hands after you washed them? 🞏 Yes 🞏 No 🞏 DK

***If yes,*** did you use it? 🞏 Yes 🞏 No 🞏 DK

1. Was hand sanitizer available? 🞏 Yes 🞏 No 🞏 DK

***If yes,*** did you/your child use it after contact with the

animals or their environment? 🞏 Yes 🞏 No 🞏 DK

1. Did you/your child eat, drink, chew, or smoke anything while with the animals? 🞏 Yes 🞏 No 🞏 DK
2. Did you/your child remove your shoes once you got home? 🞏 Yes 🞏 No 🞏 DK
3. Did you/your child change your clothes once you got home? 🞏 Yes 🞏 No 🞏 DK
4. *Interviewer: did case admit to drinking raw milk on surveillance interview?*

🞏 Yes **(*complete unpasteurized milk follow up questionnaire)***

🞏 No **(*re-ask if they drink unpasteurized milk. If yes, complete unpasteurized milk follow up)***

1. Would you be interested in receiving educational materials about farm animals and your illness?

🞏 Yes 🞏 No

🞏 Email: _____________________________________

🞏 Home address *(confirm address from CRF)*

*That concludes our interview, thank you for speaking with me. Do you have any questions?*

Comments: ___________________________________________________________________________

_____________________________________________________________________________________________

______________________________________________________________________________________________
