# Supplementary material for: Animal agriculture exposures among Minnesota residents with zoonotic enteric infections, 2012–2016
Source: Epidemiol Infect. 2020 Mar 16;148:e55. doi: 10.1017/S0950268819002309 (PMC7078579; doi:10.1017/S0950268819002309)
Supplement: Supplementary file 1 [file S0950268819002309sup001.zip › Klumb Supplementary Figure S2.docx]

*Epidemiology and Infection*

Animal Agriculture Exposures among Minnesota Residents with Zoonotic Enteric Infections, 2012-2016

C. A. Klumb, J. M. Scheftel, K. E. Smith

Supplementary Material

**Figure S2: Tier 1 questionnaire – Live and/or work on a farm**

**UMASH Interview**

**Tier 1: Cases who lived and/or work on a food animal farm**

Patient’s Name (last, first): ________________________________________ DOB: ___/___/___

Parent’s Name (if child): __________________________________________ Onset date: ___/___/___

Infection: ___________________________________ Phone #: __________________________________

**Farm Demographics:** First I’d like to ask you some basic questions about the farm & your/your child’s role.

1. What is your position on the farm? 🞏 Owner/co-owner 🞏 Family member 🞏 Employee

🞏 Other: ____________________________

1. Does the farm grow crops in addition to raising animals? 🞏 Yes 🞏 No 🞏 DK
2. Is this a full-time or part-time operation? 🞏 Full-time 🞏 Part-time

**If part-time,** did the farm produce at least $1,000 worth of product in the last year? 🞏 Yes 🞏 No 🞏 DK

1. Do you live on the farm? 🞏 Yes 🞏 No

**If yes,** how many people live on the farm? _________ How many people are under 5 years of age? _____

**If no,** where is the farm located? Town/city:_____________________ County:_____________________

1. How long have you lived or worked on this farm? ______ 🞏 Years 🞏 Months
2. Have you ever lived or worked on another farm? 🞏 Yes 🞏 No
3. How many people work on the farm? _________

*(Interviewers: ask them to include themselves if they indicated chores on surveillance interview)*

Family Members ______ # < 18 y/o ____

Non-family Members _____ # < 18 y/o ____

1. Prior to your illness, how many hours per week were you/your child typically working on the farm or doing chores?

🞏 0 🞏 31 - 40

🞏 1 - 10 🞏 41 - 50

🞏 11 - 20 🞏 51 - 60

🞏 21 - 30 🞏 > 60

1. How much work/school or daycare did you/your child miss due to illness?

🞏 NA 🞏 2 - 3 days _______________________

🞏 None 🞏 4 - 7 days

🞏 <1 day 🞏 > 7 days

🞏 1 day

**Animal Inventory and Animal Contact**: Next I would like to ask you about the type of animals on the farm and how much contact you had with them in the 7 (14) days prior to your illness. (*Interviewer: If person doesn’t know how many animals ask them to give a ballpark estimate <10, >10, >100. Fraction of hours are ok for time in animals’ environment and contact with animals)*

1. a) Does the farm have beef cattle? 🞏 Yes 🞏 No

**If yes,** what type of beef cattle operation is being run? (*please record number of head per type*)

| Type | Cows | Replacement  Heifers | Unweaned (Calves at side) | Stockers/ Backgrounders | Cattle on Feed (to be finished) | Total |
| --- | --- | --- | --- | --- | --- | --- |
| 🞏 Cow-Calf |  |  |  |  |  |  |
| 🞏 Stocker/Backgrounder |  |  |  |  |  |  |
| 🞏 Feedlot |  |  |  |  |  |  |
| 🞏 All classes (cow-calf to finish) |  |  |  |  |  |  |
| 🞏 Other ______________ |  |  |  |  |  |  |

b) In the 7 (14) days prior to your illness, did you have contact with the animals’ environment (i.e. barn, pens)? 🞏 Yes 🞏 No

- - - 1. Please estimate the number of hours per day _________

c) In the 7 (14) days prior to your illness, did you have any physical contact with the animals (i.e. handling animals)? 🞏 Yes 🞏 No

1. Please estimate the number of hours per day _________
2. a) Does the farm have dairy cattle? 🞏 Yes 🞏 No

**If yes,** please tell us how many head of the following you currently have on your dairy farm.

| Milking Cows | Dry Cows | Replacement Heifers (post weaning to prefreshening) | Dairy Steers | Bottle/Pail Calves (unweaned) | Total |
| --- | --- | --- | --- | --- | --- |
|  |  |  |  |  |  |

1. In the 7 (14) days prior to your illness, did you have contact with the animals’ environment (i.e. barn, pens)? 🞏 Yes 🞏 No

i. Please estimate the number of hours per day _________

1. In the 7 (14) days prior to your illness, did you have any physical contact with the animals (i.e. handling animals)? 🞏 Yes 🞏 No

i. Please estimate the number of hours per day ________

1. a) Does the farm have swine? 🞏 Yes 🞏 No

**If yes,** what type of swine operation is being run? *(please record number of head per type of swine)*

| Type | Sows | Replacement Gilts | Boars | Weaned/  Nursery Pigs | Growers | Finishers | Total |
| --- | --- | --- | --- | --- | --- | --- | --- |
| 🞏 Breeding, Gestation, Farrowing |  |  |  |  |  |  |  |
| 🞏 Farrow-to-Finish |  |  |  |  |  |  |  |
| 🞏 Wean-to-Finish |  |  |  |  |  |  |  |
| 🞏 Boar Stud |  |  |  |  |  |  |  |
| 🞏 Nursery |  |  |  |  |  |  |  |
| 🞏 Finisher |  |  |  |  |  |  |  |

1. In the 7 (14) days prior to your illness, did you have contact with the animals’ environment (i.e. barn, pens)? 🞏 Yes 🞏 No

i. Please estimate the number of hours per day _________

1. In the 7 (14) days prior to your illness, did you have any physical contact with the animals (i.e. handling animals)? 🞏 Yes 🞏 No

i. Please estimate the number of hours per day _________

1. a) Does the farm have sheep? 🞏 Yes 🞏 No

**If yes,** what type of sheep operation is being run? *(please record number of head per type)*

| Type | Rams | Ewes | Lambs | Total |
| --- | --- | --- | --- | --- |
| 🞏 Milking Sheep |  |  |  |  |
| 🞏 Meat/Wool |  |  |  |  |
| 🞏 Feedlot |  |  |  |  |

1. In the 7 (14) days prior to your illness, did you have contact with the animals’ environment (i.e. barn, pens)? 🞏 Yes 🞏 No

i. Please estimate the number of hours per day _________

1. In the 7 (14) days prior to your illness, did you have any physical contact with the animals (i.e. handling animals)? 🞏 Yes 🞏 No

i. Please estimate the number of hours per day _________

1. a) Does the farm have goats? 🞏 Yes 🞏 No

**If yes,** what type of goat operation is being run? *(please record number of head per type)*

| Type | Bucks | Does | Yearlings | Kids | Wethers* | Total |
| --- | --- | --- | --- | --- | --- | --- |
| 🞏 Dairy Goats |  |  |  |  |  |  |
| 🞏 Meat Goats |  |  |  |  |  |  |
| 🞏Angora Goats |  |  |  |  |  |  |
| 🞏 Other:  ___________ |  |  |  |  |  |  |

*castrated male sheep

1. In the 7 (14) days prior to your illness, did you have contact with the animals’ environment (i.e. barn, pens)? 🞏 Yes 🞏 No

i. Please estimate the number of hours per day _________

1. In the 7 (14) days prior to your illness, did you have any physical contact with the animals (i.e. handling animals)? 🞏 Yes 🞏 No

i. Please estimate the number of hours per day ________

1. a) Does the farm have poultry? 🞏 Yes 🞏 No

*If yes, is it backyard/recreational poultry only?* 🞏 Yes 🞏 No

**If yes,** what type of poultry operation is being run? *(please record number of head per type)*

| Type | Hatchery | Breeder | Pullets | Poults | Layer | Meat  (broiler/turkey) | Other | Total |
| --- | --- | --- | --- | --- | --- | --- | --- | --- |
| 🞏 Chickens |  |  |  |  |  |  |  |  |
| 🞏 Turkeys |  |  |  |  |  |  |  |  |
| 🞏 Game Birds  ___________ |  |  |  |  |  |  |  |  |
| 🞏 Waterfowl  ___________ |  |  |  |  |  |  |  |  |

1. In the 7 (14) days prior to your illness, did you have contact with the animals’ environment (i.e. barn, pens)? 🞏 Yes 🞏 No
   - - 1. Please estimate the number of hours per day _________
2. In the 7 (14) days prior to your illness, did you have any physical contact with the animals (i.e. handling animals)? 🞏 Yes 🞏 No
3. Please estimate the number of hours per day _________
4. Do you have any other animals on the farm? (e.g. horses, cats, dogs, cervids, etc.) 🞏 Yes 🞏 No

[Please describe species and number]

________________________________________________________________________________________________________________________________________________________________________________________________________________________________________________________________________________________________________________________________________________

**Activities/job duties and personal protective equipment (PPE) questions**: I would like to ask you about your/your child’s specific job duties or chores, as well as the use of personal protective equipment on the farm in the 7 (14) days prior to getting sick.

Date 7(14) days prior to onset ___/___/___

1. Do you/your child do any chores or ever help with the animals? 🞏 Yes 🞏 No

***If no, skip down to question 51.***

1. When working on the farm, how often did you/your child wear barn boots or shoes?

Always Frequently Sometimes Rarely Never

1. When working on the farm, how often did you/your child wear dedicated work or chore clothing?

Always Frequently Sometimes Rarely Never

1. When working on the farm, how often did you/your child wear gloves?

Always Frequently Sometimes Rarely Never

**Did you/your child do any of the following?** ***(Interviewer: If no, make a big “x” through the task)***

| **Daily Management Tasks** | | | | | | |
| --- | --- | --- | --- | --- | --- | --- |
|  | **Cattle** | **Swine** | **Sheep** | **Goat** | **Poultry** | **Other:___________________** |
| 1. Feed adult animals |  |  |  |  |  |  |
| Comments: | | | | | | |
| - What type of PPE do you use when you doing this?   🞏 Gloves 🞏 Boots (leather, rubber) 🞏 Coveralls (everyday, waterproof)  🞏 Other: __________________ | | | | | | |
|  | | | | | | |
|  | **Cattle** | **Swine** | **Sheep** | **Goat** | **Poultry** | **Other:____________________** |
| 1. Feed juveniles (calves etc.) |  |  |  |  |  |  |
| Comments: | | | | | | |
| - What type of PPE do you use when you doing this?   🞏 Gloves 🞏 Boots (leather, rubber) 🞏 Coveralls (everyday, waterproof)  🞏 Other: __________________ | | | | | | |
|  | | | | | | |
|  | **Cattle** | **Swine** | **Sheep** | **Goat** | **Poultry** | **Other:_____________________** |
| 1. Clean or touch the animals’ water or water container |  |  |  |  |  |  |
| Comments: | | | | | | |
| - What type of PPE do you use when you doing this?   🞏 Gloves 🞏 Boots (leather, rubber) 🞏 Coveralls (everyday, waterproof)  🞏 Other: __________________ | | | | | | |
|  | | | | | | |
|  | **Cattle** | **Swine** | **Sheep** | **Goat** | **Poultry** | **Other:_____________________** |
| 1. Clean/scrape pens/stalls/cages |  |  |  |  |  |  |
| - What type of PPE do you use when you doing this?   🞏 Gloves 🞏 Boots (leather, rubber) 🞏 Coveralls (everyday, waterproof)  🞏 Other: __________________   - **Do you power wash the walls or floors? 🞏 Yes 🞏 No** - *(If yes)*What type of PPE do you use when you power wash walls or floors?   🞏 Gloves 🞏 Boots (leather, rubber) 🞏 Coveralls (everyday, waterproof)  🞏 Face shield 🞏 Mask (work, dust) 🞏 Goggles 🞏 N 95 respirator (poultry)  🞏 Other: __________________   - **Do you dry clean (use air to remove dust and debris)?** **🞏 Yes 🞏 No** - *(If yes)*What type of PPE do you use when dry clean?   🞏 Gloves 🞏 Boots (leather, rubber) 🞏 Coveralls (everyday, waterproof)  🞏 Face shield 🞏 Mask (work, dust) 🞏 Goggles 🞏 N 95 respirator (poultry)  🞏 Other: __________________ | | | | | | |
| Comments: | | | | | | |
|  | | | | | | |
|  | **Cattle** | **Swine** | **Sheep** | **Goat** | **Poultry** | **Other:____________** |
| 1. Bed pens/add litter |  |  |  |  |  |  |
| - What type of PPE do you use when you bed the animals’ pens?   🞏 Gloves 🞏 Boots (leather, rubber) 🞏 Coveralls (everyday, waterproof) 🞏 Mask (work, dust)  🞏 Other: __________________   - What type of material do you re-bed the pens with?   🞏 Straw 🞏 Solid-separated manure 🞏 Methane-digested manure 🞏 Sawdust | | | | | | |
| Comments: | | | | | | |
|  | | | | | | |

|  | **Cattle** | **Swine** | **Sheep** | **Goat** | **Poultry** | **Other:____________** |
| --- | --- | --- | --- | --- | --- | --- |
| 1. Handle, load, or spread manure |  |  |  |  |  |  |
| - What type of PPE do you use when you handle manure?   🞏 Gloves 🞏 Boots (leather, rubber) 🞏 Coveralls (everyday, waterproof) 🞏 Face shield  🞏 Mask (work, dust) 🞏 N 95 respirator (poultry)  🞏 Other: __________________ | | | | | | |
| Comments: | | | | | | |
|  | | | | | | |
|  | **Cattle** | **Swine** | **Sheep** | **Goat** | **Poultry** | **Other:____________** |
| 1. Milk |  |  |  |  |  |  |
| - What type of PPE do you use when you milk animals?   🞏 Gloves 🞏 Rubber boots 🞏 Apron 🞏Coveralls  🞏 Other: __________________ | | | | | | |
| Comments: | | | | | | |
|  | | | | | | |
|  | **Cattle** | **Swine** | **Sheep** | **Goat** | **Poultry** | **Other:____________** |
| 1. Collect/handle/wash eggs |  |  |  |  |  |  |
| - What type of PPE do you use when you handling eggs?   🞏 Gloves 🞏 Boots (leather, rubber) 🞏 Coveralls (everyday, waterproof)  🞏 Other: __________________ | | | | | | |
| Comments: | | | | | | |
|  | | | | | | |
| **Reproductive Health** | | | | | | |
|  | **Cattle** | **Swine** | **Sheep** | **Goat** | **Poultry** | **Other:____________** |
| 1. Help with the birthing process |  |  |  |  |  |  |
| - What type of PPE do you use when you help with the birthing process?   🞏 Plastic sleeves 🞏 Boots (leather, rubber) 🞏 Apron/ gown 🞏Coveralls (everyday, waterproof)  🞏 Gloves 🞏Other: __________________ | | | | | | |
| Comments: | | | | | | |
|  | | | | | | |
|  | **Cattle** | **Swine** | **Sheep** | **Goat** | **Poultry** | **Other:__________________** |
| 1. Rectal palpations (AI, pregnancy check) |  |  |  |  |  |  |
| - What type of PPE do you use when you perform rectal palpations?   🞏 Gloves 🞏 Boots (leather, rubber) 🞏 Coveralls (everyday, waterproof)  🞏 Other: __________________ | | | | | | |
| Comments: | | | | | | |

|  | | | | | | | | | | | | |
| --- | --- | --- | --- | --- | --- | --- | --- | --- | --- | --- | --- | --- |
|  | **Cattle** | | | **Swine** | | **Sheep** | | **Goat** | | **Poultry** | **Other:____________________** | |
| 1. Semen collection/   insemination |  | | |  | |  | |  | |  |  | |
| - What type of PPE do you use when you perform rectal palpations?   🞏 Gloves 🞏 Boots (leather, rubber) 🞏 Coveralls (everyday, waterproof)  🞏 Other: __________________ | | | | | | | | | | | | |
| Comments: | | | | | | | | | | | | |
|  | | | | | | | | | | | | |
| **Preventative Health Tasks** | | | | | | | | | | | | |
|  | | **Cattle** | **Swine** | | **Sheep** | | **Goat** | | **Poultry** | | **Other:____________** | |
| 1. Chute work (trimming hooves, deworming, dehorning, etc.) | |  |  | |  | |  | |  | |  | |
| - What type of PPE do you use when you do chute work?   🞏 Gloves 🞏 Boots (leather, rubber) 🞏 Coveralls (everyday, waterproof) 🞏 Other: _______________ | | | | | | | | | | | | |
| Comments: | | | | | | | | | | | | |
|  | | | | | | | | | | | | |
|  | | **Cattle** | **Swine** | | **Sheep** | | **Goat** | | **Poultry** | | **Other:____________** | |
| 1. Castration, clipping needle teeth, docking lamb’s tails | |  |  | |  | |  | |  | |  | |
| - What type of PPE do you use when you perform castrations?   🞏 Gloves 🞏 Boots (leather, rubber) 🞏 Coveralls (everyday, waterproof)  🞏 Other: __________________ | | | | | | | | | | | | |
| Comments: | | | | | | | | | | | | |
|  | | **Cattle** | **Swine** | | **Sheep** | | **Goat** | | **Poultry** | | | **Other:____________** |
| 1. Pouring | |  |  | |  | |  | |  | | |  |
| - What type of PPE do you use when you pour antiparasitic medication?   🞏 Gloves 🞏 Boots (leather, rubber) 🞏 Coveralls (everyday, waterproof)  🞏 Other: __________________ | | | | | | | | | | | | |
| Comments: | | | | | | | | | | | | |
|  | | | | | | | | | | | | |
|  | | **Cattle** | **Swine** | | **Sheep** | | **Goat** | | **Poultry** | | | **Other:____________** |
| 1. Vaccinations | |  |  | |  | |  | |  | | |  |
| - What type of PPE do you use when you perform vaccinations?   🞏 Gloves 🞏 Boots (leather, rubber) 🞏 Coveralls (everyday, waterproof)  🞏 Other: __________________ | | | | | | | | | | | | |
| Comments: | | | | | | | | | | | | |

| **Sick Animals** | | | | | | |
| --- | --- | --- | --- | --- | --- | --- |
|  | **Cattle** | **Swine** | **Sheep** | **Goat** | **Poultry** | **Other:____________** |
| 1. Treat sick animals |  |  |  |  |  |  |
| - What type of PPE do you use when you treat animals?   🞏 Gloves 🞏 Boots (leather, rubber) 🞏 Coveralls (everyday, waterproof)  🞏 Other: __________________ | | | | | | |
| Comments: | | | | | | |
|  | | | | | | |
|  | **Cattle** | **Swine** | **Sheep** | **Goat** | **Poultry** | **Other:____________** |
| 1. Remove dead animals |  |  |  |  |  |  |
| - What type of PPE do you use when you move dead animals?   🞏 Gloves 🞏 Boots (leather, rubber) 🞏 Coveralls (everyday, waterproof)  🞏 Other: __________________ | | | | | | |
| Comments: | | | | | | |
|  | | | | | | |
| **Other Tasks** | | | | | | |
|  | **Cattle** | **Swine** | **Sheep** | **Goat** | **Poultry** | **Other:____________** |
| 1. Move, load, transport animals |  |  |  |  |  |  |
| - What type of PPE do you use when you move, load or transport animals?   🞏 Gloves 🞏 Boots (leather, rubber) 🞏 Coveralls (everyday, waterproof)  🞏 Other: __________________ | | | | | | |
| Comments: | | | | | | |
|  | | | | | | |
|  | **Cattle** | **Swine** | **Sheep** | **Goat** | **Poultry** | **Other:____________** |
| 1. Slaughter/ butcher |  |  |  |  |  |  |
| - What type of PPE do you use when you slaughter or butcher animals?   🞏 Gloves 🞏 Boots (leather, rubber) 🞏 Coveralls (everyday, waterproof)  🞏 Other: __________________ | | | | | | |
| Comments: | | | | | | |
|  | | | | | | |
|  | **Cattle** | **Swine** | **Sheep** | **Goat** | **Poultry** | **Other:_____________________** |
| 1. Shear |  |  |  |  |  |  |
| - What type of PPE do you use when you shear sheep?   🞏 Gloves 🞏 Boots (leather, rubber) 🞏 Coveralls (everyday, waterproof)  🞏 Other: __________________ | | | | | | |
| Comments: | | | | | | |
|  | | | | | | |
|  | **Cattle** | **Swine** | **Sheep** | **Goat** | **Poultry** | **Other:_____________________** |
| 1. Brush or groom animals |  |  |  |  |  |  |
| - What type of PPE do you use when you brush/groom the animals?   🞏 Gloves 🞏 Boots (leather, rubber) 🞏 Coveralls (everyday, waterproof) 🞏 Other:_________________ | | | | | | |
| Comments: | | | | | | |
|  | | | | | | |
|  | **Cattle** | **Swine** | **Sheep** | **Goat** | **Poultry** | **Other:____________­­­­­­________** |
| 1. Other: |  |  |  |  |  |  |
| - What type of PPE do you use when you ______________?   🞏 Gloves 🞏 Boots (leather, rubber) 🞏 Coveralls (everyday, waterproof)  🞏 Other: __________________ | | | | | | |
| Comments: | | | | | | |

1. How often did you remove your work clothes before coming into the main part of the house?

Always Frequently Sometimes Rarely Never

1. How often did you remove your boots before coming into the house?

Always Frequently Sometimes Rarely Never

1. In the 7 (14) days prior to illness did you/your child eat, drink (including bottles), smoke, or chew anything while working with animals or were in their environment? 🞏 Yes 🞏 No

***If yes,*** what & where: _______________________________________________________________

1. Do you have access to clean water for people that is separate from the animals (e.g. water fountain, sink)?

🞏 Yes 🞏 No Source: ____________________________

1. Is there a non-animal area to eat your lunch? (e.g. break room, in your house) 🞏 Yes 🞏 No

Where: ____________________________________________

1. Are there sinks with soap available for hand washing in any of the agriculture buildings? 🞏 Yes 🞏 No

Where are the sinks located? How many? ____________________________________________________

1. a) When working or doing chores, how often do you wash your hands prior to eating, drinking, smoking or chewing? (circle one)

Always Frequently Sometimes Rarely Never

b) How often did you use soap when you wash your hands? (circle one)

Always Frequently Sometimes Rarely Never

1. How often do you use paper/cloth towels to dry your hands after you wash them? (circle one)

Always Frequently Sometimes Rarely Never

1. Is hand sanitizer or bacterial wipes available in the barn? 🞏 Yes 🞏 No

🞏 Hand sanitizer 🞏 Bacterial wipes

***If yes*,** how often do you use hand sanitizer or bacterial wipes? (circle one)

Always Frequently Sometimes Rarely Never

**Start here again if they didn’t do any chores**

1. In the 7 (14) days prior to illness did you/your child come into contact with manure in the house via dirty work clothes or boots? 🞏 Yes 🞏 No

52) Have you noticed any illness in the animals prior to or following your illness? 🞏 Yes 🞏 No

- - Animal type #1 ___________________
    1. Date of onset ____/____/_____
    2. Signs:______________________________________________________________________________________________________________________________________
    3. How many animals (specify type) were sick? ________
    4. Was the illness diagnosed by a veterinarian? 🞏 Yes 🞏 No
    5. Diagnosis ___________________
  - Animal type #2 ___________________
    1. Date of onset ____/____/_____
    2. Signs:______________________________________________________________________________________________________________________________________
    3. How many animals (specify type) were sick? ________
    4. Was the illness diagnosed by a veterinarian? 🞏 Yes 🞏 No

v. Diagnosis ___________________

**Knowledge, Attitudes, Beliefs**

1. Prior to this illness, did you know that agricultural animals can be a source of germs that can make people sick? 🞏 Yes 🞏 No

***If yes,*** were there any safety measures/activities/programs put in place to reduce the risk of people getting sick from contact with animals or their environment?

🞏 Yes 🞏 No

What safety measures did you have? _______________________________________________ 🞏 Unk

1. Have you changed any of your behaviors since becoming ill?___________________________________
2. How do you like to get health and safety information? (from your doctor, online, Ag journals, etc)

__________________________________________________________________

**Raw Milk Practices**

1. *Interviewer: did case admit to drinking raw milk on surveillance interview?*

🞏 Yes **(*complete unpasteurized milk follow up questionnaire)***

🞏 No **(*re-ask if they drink unpasteurized milk. If yes, complete unpasteurized milk follow up)***

**Educational Materials**

1. Would you be interested in receiving educational materials about farm animals and your illness?

🞏 Yes 🞏 No

Email: ___________________________________________________________

Home address *(confirm address from CRF)*

***(Offer them the center web address: umash.umn.edu)***

**Comments**

______________________________________________________________________________________________________________________________________________________________________________________________________________________________________________________________________________

*That concludes our interview. Thank you for taking the time to speak with me today. Do you have any questions?*
