# Supplementary material for: Animal agriculture exposures among Minnesota residents with zoonotic enteric infections, 2012–2016
Source: Epidemiol Infect. 2020 Mar 16;148:e55. doi: 10.1017/S0950268819002309 (PMC7078579; doi:10.1017/S0950268819002309)
Supplement: Supplementary file 1 [file S0950268819002309sup001.zip › Klumb Supplementary Figure S1.docx]

*Epidemiology and Infection*

Animal Agriculture Exposures among Minnesota Residents with Zoonotic Enteric Infections, 2012-2016

C. A. Klumb, J. M. Scheftel, K. E. Smith

Supplementary Material

**Figure S1: Food animal contact-related questions included in each pathogen specific interview**

1. During the week prior to your illness, did you live on, work on, or visit a farm? □ Y □ N

**If yes**, name, location, and dates at farm (other than home farm):

□ Live on farm

□ Work on farm: __________________________________________________ When? _____________

□ Visit farm: _____________________________________________________ When? _____________

1. Did you visit a petting zoo, educational exhibit, fair, or other venue with animals in the week before your illness? □ Y □ N

**If yes**, name/location: __________________________________________________ When? _____________

1. **If yes to questions 9 or 10**, were any of the following animals present? **If yes**, did you have any contact with them?

|  | **Home** | | | **Work** | | | **Other Farm** | | | **Petting Zoo/Exhibit** | | |
| --- | --- | --- | --- | --- | --- | --- | --- | --- | --- | --- | --- | --- |
|  | Present –  No Contact | Contact | Describe Contact | Present –  No Contact | Contact | Describe Contact | Present –  No Contact | Contact | Describe Contact | Present –  No Contact | Contact | Describe Contact |
| **Cow** | □ | □ |  | □ | □ |  | □ | □ |  | □ | □ |  |
| **Goat** | □ | □ |  | □ | □ |  | □ | □ |  | □ | □ |  |
| **Sheep** | □ | □ |  | □ | □ |  | □ | □ |  | □ | □ |  |
| **Pig** | □ | □ |  | □ | □ |  | □ | □ |  | □ | □ |  |
| **Chicken** | □ | □ |  | □ | □ |  | □ | □ |  | □ | □ |  |
| **Turkey** | □ | □ |  | □ | □ |  | □ | □ |  | □ | □ |  |
| **Other:** | □  □ | □  □ |  | □  □ | □  □ |  | □  □ | □  □ |  | □  □ | □  □ |  |
